# For Better or Worse: The Potential for Dose Limiting the On-Target Toxicity of PI 3-Kinase Inhibitors

**DOI:** 10.3390/biom9090402

**Published:** 2019-08-22

**Authors:** Christina M. Buchanan, Kate L. Lee, Peter R. Shepherd

**Affiliations:** 1 Department of Molecular Medicine and Pathology, Faculty of Medical and Health Sciences, The University of Auckland, Private Bag 92019, Auckland 1142, New Zealand; 2 Maurice Wilkins Centre for Molecular Biodiscovery, The University of Auckland, Private Bag 92019, Auckland 1142, New Zealand

**Keywords:** cancer, cell signaling, on-target drug toxicity, metabolism, PI 3-kinase inhibition

## Abstract

The hyper-activation of the phosphoinositide (PI) 3-kinase signaling pathway is a hallmark of many cancers and overgrowth syndromes, and as a result, there has been intense interest in the development of drugs that target the various isoforms of PI 3-kinase. Given the key role PI 3-kinases play in many normal cell functions, there is significant potential for the disruption of essential cellular functions by PI 3-kinase inhibitors in normal tissues; so-called on-target drug toxicity. It is, therefore, no surprise that progress within the clinical development of PI 3-kinase inhibitors as single-agent anti-cancer therapies has been slowed by the difficulty of identifying a therapeutic window. The aim of this review is to place the cellular, tissue and whole-body effects of PI 3-kinase inhibition in the context of understanding the potential for dose limiting on-target toxicities and to introduce possible strategies to overcome these.

## 1. Introduction

### 1.1. PI 3-Kinases are Essential to Life

Phosphoinositide 3-kinases (PI3Ks) play a critical role in pathways regulating cellular functions such as metabolism, growth and survival, cytoskeletal rearrangements and cell migration and are, therefore, essential to life [1]. They are also clearly implicated in cancer, immune dysfunction and overgrowth syndromes and, as such, PI3K inhibitors have been the focus of anti-cancer therapeutic developments [2]. The aim of this review is to discuss the essential nature of PI3K actions in cellular and whole-body function and place the effects of PI3K inhibition in context, highlighting the dose-limiting impacts of these therapeutics. Given their use in cancer and other pathologies, we also suggest how we can use our understanding of PI3K function in health and disease to tailor the use of PI3K inhibitors and utilize combination therapies.

### 1.2. The PI 3-Kinase Classes and their Signaling

**Figure 1:**
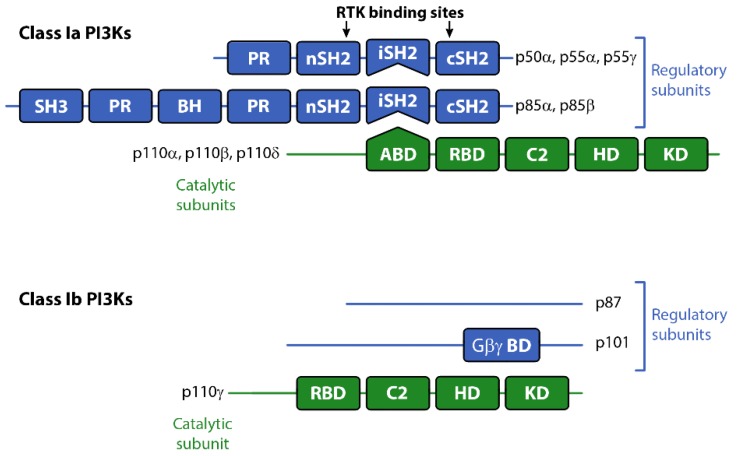
Schematic representation of Class I PI3K catalytic and regulatory subunits. Abbreviations: ABD, adaptor binding domain; BH, breakpoint cluster region homology domain (Rho-Gap-like domain); C2, C2 domain; Gβγ BD, Gβγ binding domain; HD, helical domain; KD, kinase domain; iSH2: inter-SH2 domain (p110 binding domain); PR, proline-rich domain; RBD, RAS binding domain.

There are three classes of PI3Ks grouped according to their primary lipid substrate specificity and structure (see Table 1). The Class I PI3Ks principally phosphorylate phosphatidylinositol 4,5-bisphosphate PI(4,5)P2 (aka PIP2) on the 3’OH of the inositol ring to produce PI(3,4,5)P3 (PIP3). The Class Ia PI3Ks are heterodimers consisting of a catalytic and regulatory subunit. The genes, *PIK3CA*, *PIK3CB* and *PIK3CD* code for three highly homologous 110 kDa catalytic subunits (p110α, β, or δ respectively; see Table 1). These are always found coupled to a regulatory adaptor subunit that has no catalytic activity. The genes *PIK3R1*, *PIK3R2* and *PIK3R3* code for the regulatory p85α, p85β, or p55γ proteins (see Figure 1). Two smaller forms of p85α derived from alternative promoter usage have also been identified and are termed p55α and p50α [3]. The Class Ib PI3K is also a dimer composed of a catalytic subunit, p110γ, coupled to a regulatory subunit (p101 or p84/p87PIKAP) [4,5,6]. Class II and III PI3K enzymes and a further group of kinases including mTOR and DNA-PK (sometimes referred to as Class IV PI3K) are related structurally to Class I PI3Ks [7,8,9], but are not the focus of this review. However, it should be borne in mind that structural similarities in the kinase domains of all three classes mean there is a strong potential for cross-reactivity with drugs designed to target Class I PI3Ks.

The Class I PI3Ks are acutely activated downstream of a range of growth factor receptors, cytokine receptors and by G-protein coupled receptors (GPCRs). All three Class Ia p110 catalytic subunits contain an N-terminal adaptor-binding domain (ABD; that binds to the inter-SH2 domain on p85), a Ras-binding domain (RBD), a C2 domain (putatively involved in membrane-binding), a helical domain with unknown function, and a catalytic kinase domain (see Figure 1). Growth factor receptors activate Class Ia PI3Ks via the regulatory adapter subunits, all of which contain two SH2 domains that bind directly to phosphotyrosine residues on the activated receptors and/or receptor substrates [10] (see Figure 1). This interaction localizes the p110 catalytic subunit to the plasma membrane and relieves the regulatory inhibition of p85, allowing p110 to phosphorylate PIP2 to PIP3 [11]. The Class Ib catalytic subunit p110γ together with p101, its main regulatory adapter, is mainly activated by GPCRs through interaction with Gβγ heterodimers [12]. GPCRs can also influence the activity of p110β via direct interactions with Gβγ [4]. Furthermore p110α, δ and γ can also be regulated by Ras whereas p110β interacts with the Rho subfamily of GTPases Rac and Ccd42 [13].

**Figure 2:**
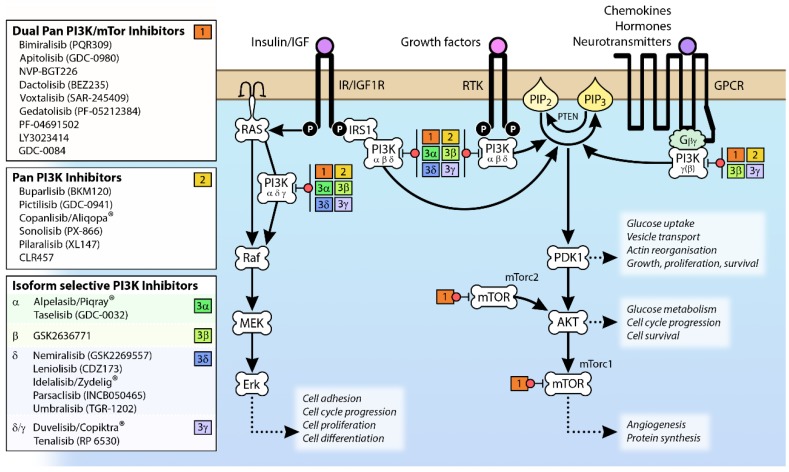
Signaling pathways activated by different isoforms of Class I PI3K and PI3K inhibitors that target specific components of these pathways. Class 1a isoforms of PI3K are attracted to the membrane by the activation of RTKs (including IR/IGF1R), while PI3Kγ (and to a lesser extent PI3Kβ) is recruited by GPCR activation. The membrane proximity of PI3K results in the phosphorylation of membrane-bound PIP2 to PIP3, which mediates the activation of downstream protein kinases involved in a wide range of cellular processes. The various pan PI3K and isoform-specific inhibitors listed are ≥Phase 2 clinical trial according to www.clinicaltrials.gov (accessed August 2019). Abbreviations: AKT, Protein kinase B; Erk, extracellular signal-regulated kinase; GPCR, G-protein coupled receptor; IGF1R, insulin-like growth factor 1 receptor; IRS1, insulin receptor; MEK, MAPK/ERK kinase; mTOR, mammalian target of rapamycin; mTorc1, mammalian target of rapamycin complex 1; mTorc2, mammalian target of rapamycin complex 2; PDK1, 3-phosphoinositide-dependent protein kinase 1; PI3K, phosphoinositide 3-kinase; PIP2, phosphatidylinositol (4,5)-bisphosphate; PIP3, phosphatidylinositol (3,4,5)-triphosphate; PTEN, phosphatase and tensin homologue; RTK, receptor tyrosine kinase.

Once produced, PIP3 initiates a range of intracellular signaling events, largely by binding to PH domains contained in certain signaling proteins. The activation of Akt is a particularly important consequence of increases in PIP3 levels. Crosstalk also exists between the Ras/Raf/MEK/Erk pathway and PI3K (see Figure 2). The levels of cellular PIP3 are tightly controlled and PI3K-dependent signaling is terminated by the dephosphorylation of PIP3, carried out by the tumor suppressor phosphatase and tensin homologue (PTEN) and SH2-domain containing inositol-5-phosphatase (SHIP), generating PI(4,5)P2 and PI(3,4)P2, respectively [14]. In this way, many signaling systems converge to effect essential cellular processes through the PI3K pathway (see Figure 2).

## 2. Tissue Distribution and Biological Roles of Class I PI3-Kinases

**Figure 3:**
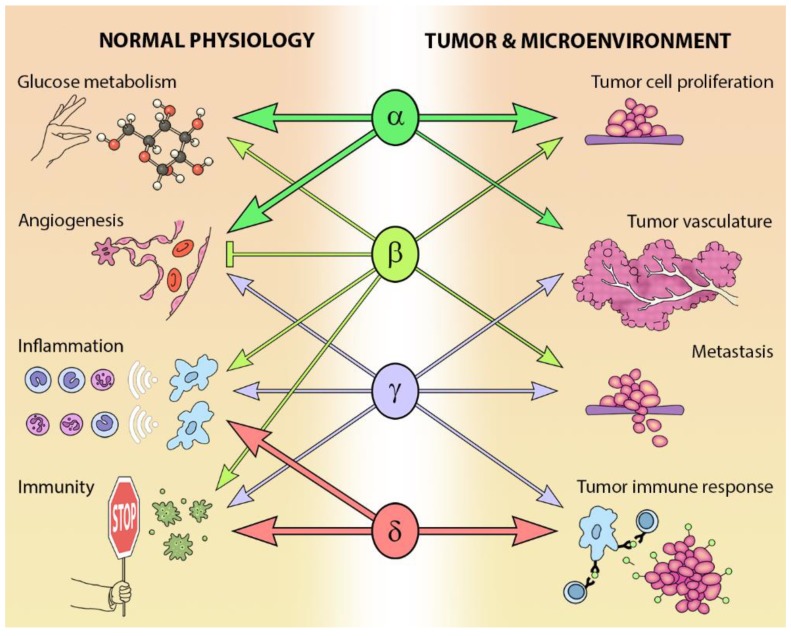
Isoform-specific roles of Class I PI3Ks in normal and cancer physiology.

The different Class I PI3K isoforms vary in their tissue distribution and impact on normal physiology (see Figure 3). PI3Kα and β are widely distributed in mammalian tissues, whereas PI3Kδ and γ are mainly, but not exclusively, expressed in blood cells and their precursors [15] (see Table 1). Due to the ubiquitous expression and central role that PI3Kα and PI3Kβ play in physiology, it is not surprising that *PIK3CA*^−/−^ mice are embryonic lethal [16] and *PIK3CB*^−/−^ mice partially embryonic lethal [17,18]. Kinase dead homozygous p110αD933A knock-in mutants are also embryonic lethal by E12.5, as a result of dysfunctional vascular development [19]. Crossing *PIK3CA*^+/−^ and *PIK3CB*^+/−^ mice results in the expected Mendelian ratio of offspring, indicating that the expression of one allele of each isoform is sufficient for normal development [20].

While PI3Kδ is mainly found in leukocytes [15], this isoform has been reported in neurons [21] and cancer cells of non-leukocyte origin, such as breast cancer cells and melanoma [15]. PI3Kδ kinase-dead knock-in mice are viable and their phenotype is restricted to defects in immune signaling and response [22,23,24].

In addition to a wide distribution in hematopoietic tissues, *PIK3CG* is also expressed in a limited number of other tissues including the heart and endothelium [25,26], as well as tumors including cancer of the pancreas and breast [27,28,29]. As might be expected from its expression profile, germ-line deletion of *PIK3CG* results in defects in innate immune and inflammatory responses [26,30,31,32,33]. These defects are relatively well tolerated and *PIK3CG*^−/−^ mice exhibit normal viability, fertility and longevity [30,31,32].

## 3. PI3-Kinase in Cancer

**Figure 4:**
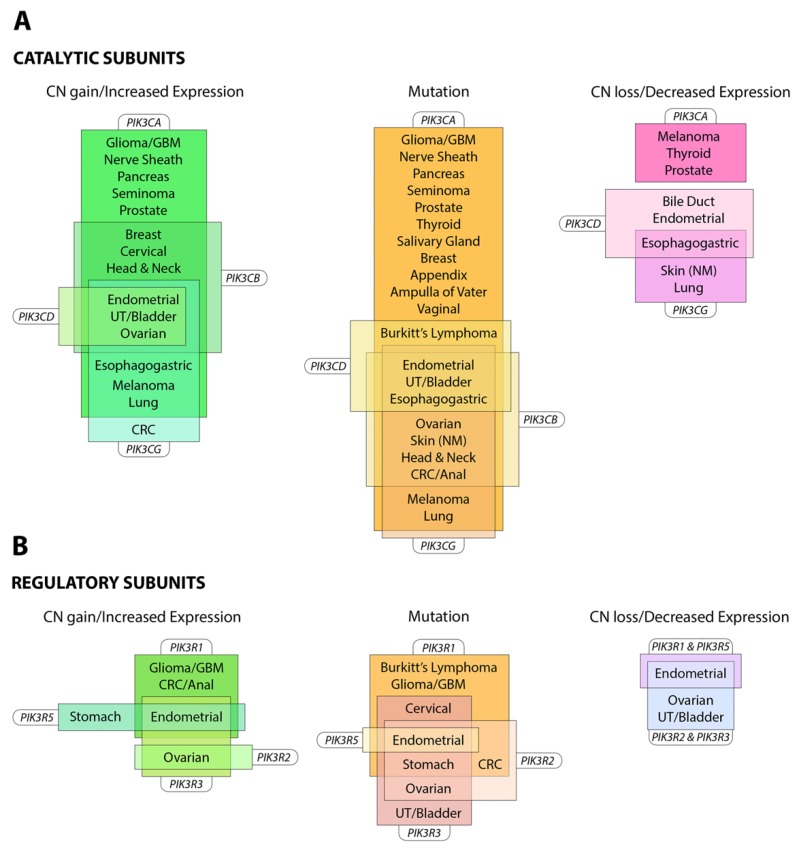
Alterations of Class I PI3K in cancer. Alterations in expression, copy number and protein sequence in (**A**) catalytic subunits and (**B**) regulatory subunits reported in the named cancers (≥5% frequency). Information summarized from www.cbioportal.org (accessed July 2019). Abbreviations: CRC, colorectal cancer; GBM, glioblastoma; NM, non-melanoma; UT, urinary tract.

The PI3K pathway is commonly dysregulated in cancer and different isoforms have varied involvement in tumors and the tumor microenvironment (see Figure 3). *PIK3CA* is reported to be one of the most commonly mutated oncogenes in human cancer [34]. Increased copy number and/or overexpression in the Class I PI3Ks also contributes to hyper-activation of the PI3K pathway in many cancers [35,36,37,38,39] (see Figure 4). The hotspot mutations E545K and H1047R in the helical and kinase domains of p110α (*PIK3CA* exons 9 and 20) were first described by Samuels et al. in 2004 [40,41] and these remain a focus of oncogenic research. More recently, a number of next-generation sequencing programs have identified a range of rarer mutations in the other Class I isoforms (data available at www.cbioportal.org) [42,43], with the most frequent being the hotspot mutation D1067A/V/Y occurring at the C-terminus of p110β [44], while another C-terminal mutation in p110β (E1051G/K) has also been described in several cancers. Multiple mutations have also been recently described in *PIK3CD* (N334K, E525K, R821C/H, E1021K) [45,46] and in the accessory domains of *PIK3CG* (R544*/P/Q) and *PIK3CB* (R604G/P). While numerous mutations causing amino acid changes have been found in p110β, δ and γ, these are far less common than mutations in p110α [39,41,42] and the impact of these mutations on enzyme activity is poorly understood.

The importance of the activating mutations in *PIK3CA* is further highlighted by the identification of gain-of-function *PIK3CA* mutations in a range of human tissue overgrowth syndromes [47,48,49,50]. Common activating mutations include those in the catalytic and helical domains of p110α that have been previously associated with oncogenic transformation (H1047L, H1047R, E542K, E543K and C420R), as well as a novel syndrome-specific mutation p110α R115P [49]. The PI3Kα E545K mutation has also been repeatedly implicated in overgrowth of the brain [51] along with other mutations in the PI3K pathway (*AKT3* c.49C>T p. E17K and *MTOR* c.4448C>T p.C1483Y).

Mutations also occur in the genes for the regulatory subunits *PIK3R1, PIK3R2, PIK3R3* and *PIK3R5* (see Figure 4) [52,53,54]. These result in elevation of the lipid kinase activity and oncogenic transformation—primarily through activation of p110α [55,56]. This is consistent with the theory that these mutations in p85 weaken the inhibitory interaction between p85α and p110α while preserving the stabilizing/activating interaction between p85α, SH2 and adapter-binding domain of p110α [57]. Inherited mutations have also been identified in p85 that reduce its ability to transduce signals from growth factor receptors to p110, causing reductions in insulin signaling and an insulin-resistant phenotype [58].

The observation that the PI3K pathway is activated in many different cancers through mutation and/or overexpression (see Figure 4) has driven the development of a range of PI3K pathway inhibitors as potential cancer therapies [59] (see Figure 2). However, the clinical efficacy of single agents targeting PI3K, AKT and mTOR has been limited [60]; indeed, phase 1b clinical trials BEZ 235 were halted due to “significant toxicity with no objective responses” [61].

The availability of PI3K crystal structures [62,63,64,65] has resulted in the supersession of early, pan-specific PI3K inhibitors by potent compounds targeting specific Class I isoforms in an effort to limit side effect profiles. There are signs this approach is more successful, because while one pan-PI3K inhibitor (copanlisib/Aliqopa®) has been approved for relapsed follicular lymphoma [66], several isoform-specific inhibitors are now on the market; the PI3Kδ inhibitor idelalisib (Zydelig®) has been approved to target chronic lymphocytic leukemia (CLL) [67,68], the dual PI3Kδ/γ inhibitor duvelisib (Copiktra®) for relapsed or refractory CLL or small lymphatic lymphoma [69] and the PI3Kα inhibitor alpelisib (Piqray®) for HR+/HER2− advanced breast cancers with *PIK3CA* mutations [70].

While non-specific off-target actions of drugs always have the potential to negatively affect the therapeutic window, the pleiotropic importance of PI3K in cellular processes means that on-target effects of PI3K inhibitors also significantly limit their therapeutic use (see Table 2). This review will present the cellular, tissue and whole-body effects of PI 3-kinase inhibition in the context of understanding the potential for dose limiting on-target toxicities and introduce possible strategies to overcome these.

## 4. Targets of PI3-Kinase Inhibitors

### 4.1. Effects of PI3K Attenuation on Whole Body Glucose Metabolism

In general, the inhibition of PI3K has been observed to counteract the effects of nutritional excess and results in normoglycemia, reduced fatty liver, and reduced adiposity [71]. The actions of insulin on glucose metabolism are mediated by PI3K [14] so it is not surprising that in clinical trials, hyperglycemia was a common effect of many early pan PI3K inhibitors [72,73,74,75,76,77], as well as dual-specificity PI3K/mTOR inhibitors [78] and PI3Kα-specific inhibitors [79,80]. Mild hyperglycemia has also been observed after administration of the PI3Kβ inhibitor GSK2636771 [81]. Indeed, the metabolic impact of inhibitors targeting the PI3K/Akt/mTOR (PAM) pathway has led to management guidance being provided by the PAM Task Force convened by the National Cancer Institute Investigational Drug Steering Committee [82].

The metabolic effects of short- and long-term PI3K attenuation have been studied preclinically in mice either by the genetic knock-down of PI3K activity [83,84], or pharmacologic inhibition [85,86]. Interestingly, both groups observed that the short-term effect of PI3Kα knock-down results in greater metabolic disturbance than chronic PI3K reduction (summarized in Table 3) [83,84,85,86]. This strongly suggests that some degree of feedback compensation is occurring over time, a finding that has been supported by a recent study that actively targeted this feedback loop using dietary and pharmacological strategies to improve the efficacy of PI3K inhibition in the treatment of tumor-bearing mice [87]. It is of note that the pharmacologic inhibition of PI3Kβ, δ and γ had much less of an effect on glucose metabolism, indicating that metabolic disturbance will be of most relevance with drugs targeting PI3Kα [85,86]; however, the genetic knock-out of PI3Kγ has been shown to ameliorate the development of diet-induced insulin resistance and Type 2 diabetes [88]. The many sites at which PI3K inhibition could impact on glucose homeostasis are discussed below.

#### 4.1.1. The Role of PI3K in Insulin Secretion

The regulated secretion of insulin from β-cells is a key process for regulating glucose homeostasis; however, there are contradictory findings regarding the role of PI3K in insulin secretion. Acute studies (30–90 minutes of inhibitor exposure) using β-cell lines or isolated islets indicate that PI3K inhibition increases insulin secretion [89,90,91,92,93], with the majority of this effect being due to PI3Kα-mediated effects [94]. These *in vitro* studies align with the increased insulin secretion and islet hyperplasia observed in kinase-dead p110βK805R/K805R mice [17], however, PI3Kβ inhibition by shRNA in cultured β-cells has been shown to result in a decrease in insulin secretion [94]. It is further proposed that PI3Kγ signaling via GPCRs controls constitutive insulin secretion by coordinating intracellular processes of trafficking and secretion [95,96] and mediating GIP-induced insulin secretion [97]. This picture is further complicated by feedback systems in responsive metabolic systems; hyperglycemia resulting from inhibition of PI3K is corrected through increased insulin secretion which, in turn, activates PI3K/mTOR signaling, reducing the impact of the primary PI3K inhibition [87]. This feedback response would be blunted in obese patients exhibiting underlying insulin resistance and those with diabetes where the insulin secretory capacity is compromised.

#### 4.1.2. Central Metabolic Effects on Appetite

Appetite loss is already a serious issue in the clinical management of cancer patients [98], so it is important to understand how anti-cancer drugs will affect appetite. Decreased appetite and/or dysgeusia (taste distortion) has been observed after the clinical administration of pan PI3K inhibitors [73,75,77,99], a dual PI3K/mTOR inhibitor (BEZ235) [78], as well as PI3Kα-isoform-specific inhibitors [79,80] and the PI3Kβ inhibitor GSK2636771 [81]. This negative effect on appetite would not necessarily have been anticipated, as several preclinical studies indicated that pan-PI3K inhibitors block leptin and insulin signaling in the hypothalamus, slightly increasing food uptake [100,101,102,103,104]. Furthermore, the tissue-specific knock-out of PI3Kβ in hypothalamic pro-opiomelanocortin (POMC)-expressing neurons resulted in leptin resistance and a diet-induced increase in adiposity [105]. One acute inhibitor dosing study that does align with the clinical trial results showed that dual PI3K/mTOR inhibitor BEZ235, PI3Kα inhibitor PIK75 and DNA-PK/PI3Kα/mTOR inhibitor PI-103 all significantly decreased food intake, while inhibitors of PI3Kβ, γ and δ had no effect [86]. Reassuringly, these acute effects on food intake were not sustained with long-term dosing, again implying that some degree of compensation is occurring [85].

#### 4.1.3. Metabolic Effects in Muscle and Adipose Tissue

Inhibition with PI3Kα-specific inhibitors (PIK-90, PI-103, or PIK-75) blocked insulin-stimulated glucose uptake in vivo [106], resulting in insulin resistance. This is supported by findings that PI3Kα is the dominant isoform required to mediate insulin and IGF-I signal transduction in muscle and adipocytes, which are key organs in the regulation of glucose disposal [84,106,107]. Increased fat mass has been demonstrated in young PI3Kα mutant mice [84]. However, this is compensated for in chronic administration and older PI3Kα mutant mice, which show a lean phenotype with reduced adiposity [83,85]. Inhibition of PI3Kα using PIK-75 also completely abolished adipocyte differentiation as assessed by morphology, transcript and protein levels of adipocyte markers [108]. Therefore, pan-inhibitors (LY294002 and wortmannin), which attenuate insulin-stimulated glucose uptake in muscle and adipocytes in a dose-dependent manner [109,110], most likely exert these actions through their inhibition of PI3Kα.

Evidence has emerged that indicates a role for PI3Kβ in skeletal muscle differentiation and myogenesis [111]. This observation correlates with the finding that PI3Kβ levels are significantly reduced in the muscle and adipose of men born with low birthweight [112,113], a group who have been identified as being susceptible to an increased risk of developing diabetes [114]. However, the inhibition of PI3Kβ has not been shown to have any effect on insulin-induced Akt phosphorylation in muscle or adipose [106] or adipocyte differentiation [108].

It has also been shown that PI3Kγ acts within adipose tissue to promote fat mass gain. PI3Kγ KO mice are leaner than their control littermates; they have increased energy expenditure and, therefore, show reduced fat gain, despite normal caloric intake. Furthermore, *PIK3CG*KD/KD mice fed a high-fat diet exhibit less weight gain and are apparently protected from insulin resistance and diet-induced steatosis [115].

#### 4.1.4. Metabolic Effects in the Liver

Insulin action in the liver is critical for maintaining normoglycemia, as glucose storage (glycogenesis), breakdown (glycolysis) and production (glycogenolysis and gluconeogenesis) in the liver are all regulated by insulin [116]. A liver-specific KO of PI3Kα results in a diabetic syndrome with decreased insulin sensitivity, impaired glucose tolerance, increased gluconeogenesis and leptinemia and decreased lipidemia [117]. In support of these genetic findings, the acute treatment of mice with PI3Kα or pan-PI3K inhibitors results in the increased gluconeogenic production of glucose from pyruvate [86]. Once again, these acute effects differ from those in a chronic model, with the long-term low-dose use of pan-PI3K inhibitors resulting in reduced liver-steatosis in mice and monkeys [118].

While the role of PI3Kα in liver metabolism appears definitive, the role of PI3Kβ in the liver remains unclear. Both genetic and pharmacologic studies exist which indicate that PI3Kβ has little effect on liver metabolism [86,117]. However, other studies have shown that mouse models lacking functional PI3Kβ either globally, or specifically in the liver, have defects in glucose metabolism [17,119,120]. Furthermore, it appears many of the cellular effects of *PIK3CB* liver-specific KO are mediated through a lipid kinase-independent function, since the impaired insulin sensitivity and glucose homeostasis were not mediated via Akt phosphorylation [119] and could be due to the alternative signaling of PI3Kβ through G-protein coupled receptors [121].

PI3Kγ has also been implicated in liver metabolism. Both PI3Kγ kinase-dead mice [115] and PI3Kγ-KO mice [88] fed a high-fat diet demonstrate a reduction in hepatic steatosis. However, a recent study has reported large changes in liver structure and function in a pancreatic neoplasia-bearing mouse model with the partial or complete knock down of PI3Kγ [122].

Taken together, these studies all indicate that disruption of the PAM pathway results in an inability for the liver to sense satiety and ultimately impacts whole-body metabolic homeostasis.

### 4.2. Effects of PI3K Attenuation in the Gut

Some of the most common side effects of the clinical use of PI3K/mTOR inhibitors are colitis, diarrhea, nausea and vomiting (see Table 2). While these are common effects for many types of drugs, the mechanism for idelalisib-induced colitis is thought to be mediated at least in part through the enhanced inflammatory response occurring in response to gut pathogens [123], and there is some evidence that points to this dose-limiting toxicity being a PI3K class effect, since PI3Ks play roles in gut immunity, motility and neuro-transmission [124].

The role of PI3K signaling in immune cells of the intestinal mucosa has been extensively reviewed [125]. In the gut, PI3K signals downstream of Toll-like receptors and T-cell receptors to mediate immune homeostasis in the face of commensal and pathogenic bacteria. Evidence suggests that PI3Kγ [125] and PI3Kδ [126,127] are important isoforms in intestinal inflammation and that deregulation of the PI3K pathway can result in inflammatory bowel disease and its associated cancers.

Interstitial cells of Cajal (ICC) are required for normal gut motility and in turn, the normal development of ICC is largely dependent upon c-Kit signaling [128]. Pan-PI3K inhibitors wortmannin and LY294002 cause the loss of ICC and the suppression of slow wave in mouse jejunal muscle strips [129]; however, the deletion of c-Kit-induced PI3K signaling (via disruption of PI3K binding to Y719F on c-Kit) was found to have no effect on the function or development of ICC in mice [128]. This latter finding indicates that c-Kit and PI3K are exerting their effects in an independent manner.

With regards to neurotransmission, PI3Kα negatively regulates the secretion of the intestinal peptide neurotensin, which stimulates GI secretion, motility and the growth of the small intestine and pancreas [130]. Furthermore, decreased PI3K activity is proposed to cause apoptosis of enteric neurons in the intestine, resulting in delayed gastric emptying and more rapid intestinal transit [131].

Therefore, mechanisms involved in PI3K-mediated effects on gut motility indicate contrasting roles. On the one hand, PI3K inhibitors have been shown to slow gut motility via the loss of ICC [129], while on the other, gut motility would be expected to increase as a result of PI3K-mediated down regulation of neurotensin [130] and the associated loss of intestinal innervation [132].

### 4.3. Effects of PI3K Attenuation in the Brain

In addition to the central effects on appetite that have already been covered in this review, pan PI3K inhibitors have also been linked to mood alterations. Wider class inhibition also commonly results in fatigue—a symptom that could have a psychological component. Certain PI3K inhibitors are known to cross the blood–brain barrier including BKM120, XL147, XL765, GDC0084, PQR309 and BEZ235 [133,134,135]. BKM120 has a depressive effect in some humans [76,135] and long-term use of PI3Kα inhibitors (BEZ235, PIK75 and PI-103) and wortmannin in mice was found to produce signs of depression [85,136], while rats treated with wortmannin and LY294002 also display learning and memory defects [137] and a reduced fear response [138]. Taken together, these data highlight the need for the psychological monitoring of patients in PI3K drug trials, particularly if they are taking drugs that are known to cross the blood–brain barrier.

### 4.4. Effects of PI3K Attenuation in Airways

Pneumonitis, or inflammation of lung tissue and alveoli, has been reported as a side effect associated with the PI3K inhibitor, idelalisib (Zydelig®), across multiple clinical trials, even causing death in a small number of patients (3/760; 0.4%) in an early clinical trial [139]. While the actual mechanism of idelalisib-induced pneumonitis remains unclear, it is now recognized that there is an increased risk of infection due to the immunomodulatory effects of this PI3Kδ inhibitor—likely mediated, at least in part, through the enhanced inflammatory response occurring in response to pathogens present in the airways [123,140,141]. It is also known that the PI3K pathway plays an important role in airway smooth muscle (ASM) development, contractility and inflammation, with many studies focusing on regulation of the PI3K pathway as a way to control asthma and chronic obstructive pulmonary disease (COPD) [142]. Therefore, there are potential benefits and risks of targeting PI3K in this tissue.

ASM hyperplasia within the bronchial wall of asthmatics is thought to be due to increased muscle proliferation, which is under the control of both the ERK and PI3K pathways. A number of mitogens acting through RTKs and GPCRs, and to a lesser-known extent, cytokine receptors, activate the PI3K and ERK signaling pathways to stimulate the proliferation of ASM [143,144]. In asthmatics, there is some evidence that it is upregulation of the PI3K (rather than the ERK) pathway that results in muscle hyperplasia [145]. PI3Kα, δ and γ all interact with RAS via their RAS-binding domain (RBD), whereas the RBD of PI3Kβ does not interact with RAS, but rather with RAC1 and CDC42 from the RHO family of GTPases. Even so, PI3Kβ is still implicated in lung pathology since mice with RBD mutant PI3Kβ are resistant to experimental lung fibrosis (a pathology linked with lysophosphatidic acid signaling through GPCRs) [119,146].

In addition to stimulating ASM growth and differentiation, the PI3K pathway is involved in ASM contractility, which in turn, is implicated in airway hyper responsiveness (AHR) and as a pro-inflammatory signaling pathway in the airway [147]. The role of eosinophils in asthma pathology has been well documented since the original observations of Huber and Koessler in 1922 [148] and it is known that PI3Kγ is essential for triggering eosinophil influx [149,150]. Furthermore, PI3Kδ has a role in regulating eosinophil trafficking and recruitment during allergic airway inflammation [151] and AHR [152].

While the role of PI3K inhibition has been thoroughly explored in relation to asthma and COPD, the severe hypersensitivity pneumonitis experienced by a small number of patients receiving idelalisib rings a note of caution [139]. Furthermore, since the idelalisib-induced pneumonitis is consistent with mTOR inhibitor-induced pneumonitis [153], this biological side-effect appears to be a rare but severe class effect. An expert panel commenting on the management of cancer patients undergoing mTOR inhibitor treatment has recommended clinical trial exclusion or close monitoring of patients with pre-existing lung disease, severe pulmonary compromise or active lung infection [154] and provides a framework for the management of patients on idelalisib, which may be adopted for broader PI3K inhibitor use [155].

### 4.5. Effects of PI3K Attenuation on Inflammation, Immunity and the Hematopoietic System

PI3K isoforms play multiple roles in the immune system and these could potentially be exploited to directly target certain leukemias (as has been done with idelalisib and duvelisib) or to modulate the actions of the highly successful immunotherapies that have been developed recently. Conversely, there is the possibility that PI3K inhibitors could negatively impact the patient’s immune system. Readers are referred to an excellent review on the roles of PI3K signaling in inflammatory and autoimmune diseases and hematological malignancies [156]; however, for completeness, critical findings will be described here.

Given the largely localized expression of PI3Kδ and γ in leukocytes [15], it is to be expected that these isoforms are the most important in immune regulation and the hematopoietic system. As mentioned previously, knocking out PI3Kδ or γ does not affect viability, fertility or longevity in mice [22,30,31,32]. However, under conditions of immune challenge, these mice exhibit deregulation of B and T cells, NK cells, dendritic and mast cells, macrophages, basophils, eosinophils and neutrophils [156].

PI3Kδ plays a key role in agonist-induced B-cell receptor (BCR) signaling [22,23,157,158,159], but agonist-independent or ‘tonic’ BCR signaling is not affected by PI3Kδ KO [160]. This is due to redundancy in the Class I PI3K family, as it has been confirmed that in the absence of PI3Kδ or PI3Kα (but not PI3Kβ), compensation occurs to promote early B-cell development in marrow and B-cell survival in the spleen [160,161]. In the absence of both PI3Kα and PI3Kδ, pre-BCR signaling failed to promote the developmental progression of B-cell progenitors [160].

PI3Kγ transduces a central pro-inflammatory signal involved in leukocyte chemotaxis, mast cell degranulation and endothelial cell activation [115] and can suppress inflammation in a variety of mouse models of disease, including atherosclerosis [33,162], rheumatoid arthritis [163], glomerulonephritis [164], anaphylaxis [165] and multiple sclerosis [166]. Although neutrophils are enriched in PI3Kδ and γ KO models, these cells also express abundant amounts of PI3Kα and β and it is thought that all Class I PI3K isoforms may contribute to GM-CSF-mediated neutrophil survival [167]. Furthermore, the global suppression of Class I PI3K activity below a certain threshold is required to abrogate this survival effect since it is not until PI3Kα, β and δ were inhibited that effects were seen [167].

Due to their hematopoietic tissue and cancer-specific expression patterns, PI3Kδ and γ inhibitors have been used to target relapsed or refractory lymphoma including, but not limited to, CLL, mantle cell lymphoma and non-Hodgkin lymphoma [67,168,169,170,171,172,173]. Unsurprisingly, these PI3Kδ and γ isoform-specific inhibitors are also noted for hematologic toxicities including anemia, thrombocytopenia, leukocytosis, hemolysis and neutropenia [168,169,173,174]. These hematopoietic toxicities are often noted in trial outcomes as common laboratory abnormalities [173,175], although none, other than neutropenia, are serious enough to be noted as a warning in the US prescribing information for idelalisib [139,176]. Furthermore, respiratory infection was observed in 20% of patients receiving idelalisib on a trial for relapsed or refractory mantle cell lymphoma [172] and PI3Kδ inhibition with AMG 319 resulted in elevated T-reg cells (>10% of CD4+); however, the T-reg cells of most patients normalized with continued treatment, indicating immune restoration [169].

Genetic and pharmacological blockade studies show that PI3K regulates the development, activation and differentiation of B- and T-cells [177]. This can have both positive and negative effects. On the positive side, PI3K inhibition can help attenuate immune response, but on the negative side, it can enhance inflammation, disrupt peripheral tolerance and promote autoimmunity [177,178]. This enhanced inflammatory response occurs in the parts of the body most exposed to pathogens (skin, airways and gut) and can exhibit strong side effects upon PI3Kδ inhibition, resulting in therapy-limiting rashes, pneumonitis and colitis [123].

Interestingly, PI3Kδ inhibition has been found to have a positive anti-inflammatory effect in ischemic brains and it has been proposed that PI3Kδ inhibition could help treat ischemic strokes [179] via a mechanism involving tumor necrosis factor-α (TNF-α). It is also suggested as a therapy for people suffering from activated PI3K-delta syndrome (APDS), who have activating mutations in *PIK3CD* [180].

### 4.6. Effects of PI3K Attenuation in the Skin

Our skin is the largest organ in our body and provides protection from pathogens, promotes thermoregulation and prevents dehydration. Therefore, while a mild skin rash might not be serious enough to be dose limiting, severe grades of rash can impact on daily living [181]. While rashes have been reported as a side effect of other targeted therapies, chemotherapy, immunotherapy, radiation therapy and stem cell transplants [181], maculopapular rash is one of the common dose-limiting toxicities reported for pan PI3K and dual PI3K/mTOR inhibitors [74,75,76,99,182] and for the PI3Kδ inhibitor idelalisib [172], but not for other isoform-specific inhibitors. Since rashes are commonly associated with multiple different therapies, the rash linked with pan PI3K inhibitor drug use is unlikely to be a class effect. Further reassurance can be derived from the fact that isoform-specific inhibitors (other than PI3Kδ) have not been reported to result in a rash. As mentioned previously, the mechanism for idelalisib-induced rash is thought to be mediated at least in part through the enhanced inflammatory response occurring in response to skin pathogens. On a positive note, where rashes are encountered as a side effect, it has been suggested that clinicians could use this effect as a pharmacodynamic biomarker for drug titration [183] in the same way rash outbreak is used to titrate the dose of EGFR inhibitors [184].

### 4.7. Effects of PI3K Chronic Attenuation in Bone

Whilst no adverse clinical effects have been noted in bone, preclinical studies show that PI3K plays a role in both osteoblasts and osteoclasts and is involved in bone formation and resorption [185], with PI3Kα being the dominant isoform in skeletal bone [186]. In osteoclasts, PI3Ks are activated by cytokines and growth factors (e.g., CSF-1, RANKL and alphavB3 integrin), resulting in osteoclast survival, development and motility [185]. PI3Kα inhibitors (BEZ-235, PIK75 and PI-103) reduce bone resorption and promote differentiation and survival of osteoblasts [185,186,187].

The effect of the long-term administration of PI3K inhibitors on various parameters of bone function in mice suggests that pan-PI3K inhibitors might be detrimental to skeletal health [86]. Smith et al. showed that the pan-PI3K inhibitors (ZSTK474, PI-103, and BEZ235) decreased bone density and either decreased or tended to decrease bone strength [86]. Furthermore, two PI3Kα inhibitors (PIK75 and A66) also reduced bone density and strength, while PI3Kβ and δ inhibitors did not show any consistent effects on bone [86]. This indicates that PI3Kα is the isoform that is most important in regulating bone mass and strength, which is consistent with findings which demonstrate that PI3Kα is by far the most prevalent Class Ia PI3K isoform expressed in bone [86,186]. These findings are supported by other studies which demonstrated that the genetic activation of skeletal PI3K signaling is associated with increased bone formation [188], while those that abrogate PI3K signaling by knockdown of Akt are accompanied by decreased bone formation and bone density [189,190,191].

While no increase in bone mass was observed using the PI3Kγ inhibitor AS252424 [86], mice lacking PI3Kγ exhibit increased bone mass and density, through the modulation of osteoclastogenesis [192].

Studies to date have not fully elucidated the mechanism(s) of the skeletal effects of PI3Kα inhibitors, however, these preclinical findings suggest that the evaluation of skeletal health (bone turnover markers and bone density) be undertaken in ongoing and planned clinical trials of pan PI3K inhibitors and PI3Kα selective inhibitors.

### 4.8. Effects of Chronic PI3K Attenuation in the Heart

PI3Ks are widely distributed throughout the cardiovascular system, with cardiac cells expressing PI3Kα, β and γ [193]. PI3Kα is essential for cardiomyocyte viability and growth mediated via PAM signaling plays an important role in cardiac hypertrophy [25,194,195,196,197]. The knockout of PI3Kα or β in cardiac myocytes (either during development or in adults) results in changes in heart structure, leading to heart failure and death [198]. PI3Kα protects against myocardial infarction [199] and regulates the expression of genes essential to maintaining cardiac structure and Z-disc alignment and signaling [200], while PI3Kα and β are essential for maintaining the organized network of T-tubules by regulating junctophilin-2 localization, which is vital for efficient Ca^2+^ induced Ca^2+^ release and ventricular contraction [198]. Preclinical studies have also shown that pharmacological PI3Kγ inhibition impacts the heart; AS605240 suppressed Akt phosphorylation in an in vivo model of myocardial infarction—decreasing inflammation and increasing cardiomyocyte apoptosis [201].

In general, it is thought that PI3Kα is the most important isoform for maintaining cardiomyocyte size, while PI3Kγ is involved in cardiac function and contractility [202,203]. PI3Kα is positively associated with heart health [204] and the loss of PI3Kα accelerates pathological ventricular remodeling and heart failure in rodent models of chronic adrenergic stimulation, primary cardiomyopathy and pressure overload [205]. It has been proposed that the use of PI3Kα inhibitors, while likely to be safe in patients with normal cardiac function, may cause cardiac dysfunction and possibly heart failure in patients with pre-existing cardiac disease [206,207]. Conversely, PI3Kγ inhibition has been associated with improved cardiac function [208,209].

## 5. Conclusions and Future Directions

The activation of the PI3K pathway in cancer has led to a huge investment in developing inhibitors targeting this pathway. Despite these intensive efforts, very few PI3K inhibitors have been approved for clinical use. As discussed above, a major factor affecting the clinical utility of these drugs has been the potential for on-target toxicities that become dose-limiting due to the fact that the PI3K pathway is so important in such a wide range of physiological and metabolic responses. Some of these on-target toxicities may eventually be clinically manageable through dietary and pharmacological strategies to control the metabolic effects [87]. Patients who are already metabolically, or immunologically challenged should be excluded from treatment [82,123,139] and biomarkers could be developed to identify further groups at risk of adverse side effects. Conversely, there may be a subset of patients with diseases that are particularly sensitive to PI3K inhibitors; in the case of cancer therapy, this is a concept known as oncogene addiction [210]. There is emerging evidence that patients with H1047R mutations in p110α respond better to lower doses of PI3K inhibitors than other tumors do [211]. Furthermore, there are promising signs that on-target toxicities can indeed be ameliorated; it has recently been shown that the PI3Kα inhibitor BYL719 (alpelasib/Piqray®) can be administered in a way that minimizes side effects while delivering clinical benefit in patients suffering from overgrowth syndromes driven by somatic *PIK3CA* mutations [212]. Alternative dosing strategies may also be successful. In this regard, it is notable that the dosing regimen for copanlisib (Aliqopa®)—the only pan-PI3K inhibitor to receive FDA approval so far—is administered intravenously once per week, rather than by oral daily dosing (routinely used to achieve the maximum tolerated dose). However, the narrow therapeutic window for PI3K inhibitors and the fact that they largely induce cytostasis rather than cell death, has seen a current trend in clinic-to-trial progression of using PI3K inhibitors at lower tolerable doses in combination with other specific inhibitors [60,213]. Combination therapies may also offer the possibility of dosing PI3K inhibitors metronomically [214] to achieve short-term synergistic effects with other agents. Another strategy for improving the tolerability of PI3K-targeted drugs could be to make PI3K inhibitor pro-drugs that are only activated in the tumor tissue. This has been achieved chemotherapeutically by adding chemical triggers to the inhibitor which make the intact pro-drug inactive; however, as these pro-drugs break apart, they release their chemotherapeutic agent to achieve high concentrations of the active drug in the tumor relative to the periphery [215]. Pro-drug strategies that take advantage of the hypoxic environment present in many tumors are a particularly attractive approach in this regard [216]. Finally, it may be possible to take advantage of the small structural differences caused by oncogenic mutations in PI3K to develop drugs that selectively target the oncogenic forms and thus spare normal signaling via PI3K.

In conclusion, PI3K inhibition is an effective strategy for targeting cancer cells and overgrowth syndromes at multiple levels, but the reality of on-target toxicity in non-tumor tissues means much work remains to be done to develop new treatment strategies if this class of drugs is ever to be used as an effective part of a chronic treatment regime for cancer or overgrowth therapies.

## Figures and Tables

**Table 1:** Gene and protein names of catalytic and regulatory subunits that make up the Class Ia and Class Ib phosphoinositide 3-kinases (PI3K) heterodimers. Also shown are the Class II and III phosphoinositide 3-kinases (PI3Ks) members, tissue distribution and the reaction catalyzed by the different classes of PI3K.

Class	Catalytic Subunits		Regulatory Subunits		Tissue Distribution	Catalytic Reaction
	Protein	Gene	Protein	Gene		
Ia	p110α	*PIK3CA*	p85α	*PIK3R1*	Ubiquitous	PI(4,5)P_2_→PI(3,4,5)P_3_
	p110β	*PIK3CB*	p85-β	*PIK3R2*	Ubiquitous	
	p110δ	*PIK3CD*	p55-γ	*PIK3R3*	Leukocytes, Neurons	
			p55-α	*PIK3R1*		
			p50-α	*PIK3R1*		
Ib	p110γ	*PIK3CG*	p101	*PIK3R5*	Leukocytes, Cardiac myocytes, Endothelium	PI(4,5)P_2_→PI(3,4,5)P_3_
			p84/p87PIKRAP	*PIK3R6*		
II	PI3K-C2α	*PIK3C2A*			Epithelium, Endothelium	PI→PI3P and PI4P→PI(3,4)P_2_
	PI3K-C2β	*PIK3C2B*			Ubiquitous	
	PI3K-C2γ	*PIK3C2G*			Hepatocytes	
III	Vps34	*PIK3C3*			Ubiquitous	PI→PI3P

**Table 2:** Clinical toxicities associated with PI3K inhibitor use. The most likely physiological target for the named toxicity is also provided.

Kinase Target	Clinical Toxicities	Physiological Target
Pan PI3K Pan PI3K/mTOR PI3Kα/β/δ/γ	Colitis/diarrhea	Gut
Pan PI3K Pan PI3K/mTOR PI3Kα	Hyperglycemia	Glucose metabolism
Pan PI3K Pan PI3K/mTOR PI3Kα/β/δ	Fatigue	Energy metabolism, Neurological
Pan PI3K	Mood alterations	Neurological
Pan PI3K Pan PI3K/mTOR PI3Kα/β/δ	Nausea/vomiting	Gut
Pan PI3K Pan PI3K/mTOR PI3Kα/β	Decreased appetite	Brain, Gut
Pan PI3K PI3Kδ	Liver dysfunction	Liver
Pan PI3K Pan PI3K/mTOR PI3Kδ	Rash	Skin
PI3Kδ	Pneumonitis/pneumonia	Airways
PI3Kδ/γ	Hematologic toxicities: anemia, neutropenia, thrombocytopenia	Hematopoietic system
PI3Kδ	Pyrexia (fever)	Unspecified
Pan PI3K	Dysgeusia	Unspecified

**Table 3:** Acute and chronic metabolic effects of PI3K abrogation in a preclinical setting.

**Acute Administration of PI3K Inhibitors (Single Dose) [86]**	**Chronic Administration of PI3K Inhibitors (22 Days Dosing) [85]**
Insulin resistant; increased gluconeogenesis, decreased glucose disposal	Insulin sensitive; normal gluconeogenesis, normal glycaemia
Decreased food intake	No effect on food intake, but decreased weight gain, fat mass, bone volume and bone strength
Decreased movement	Decreased movement
**Young PI3Kα deficient mice [84**]	**Aged PI3Kα deficient mice [83]**
Insulin resistant; hyperinsulinemic, hyperleptinemic, decreased glucose disposal	Insulin sensitive; normal glycaemia, increased longevity
Increased food intake but smaller; lower weight, length, skeletal mass, increased white adipose	No effect on food intake but males remained smaller; leaner, reduced adiposity
